# Atypical Neurogenesis in Induced Pluripotent Stem Cells From Autistic Individuals

**DOI:** 10.1016/j.biopsych.2020.06.014

**Published:** 2021-03-01

**Authors:** Dwaipayan Adhya, Vivek Swarup, Roland Nagy, Lucia Dutan, Carole Shum, Eva P. Valencia-Alarcón, Kamila Maria Jozwik, Maria Andreina Mendez, Jamie Horder, Eva Loth, Paulina Nowosiad, Irene Lee, David Skuse, Frances A. Flinter, Declan Murphy, Grainne McAlonan, Daniel H. Geschwind, Jack Price, Jason Carroll, Deepak P. Srivastava, Simon Baron-Cohen

**Affiliations:** a Autism Research Centre, Department of Psychiatry, University of Cambridge, Cambridge, United Kingdom; b Cancer Research UK Cambridge Institute, Cambridge, United Kingdom; c Department of Basic and Clinical Neuroscience, Maurice Wohl Clinical Neuroscience Institute, Institute of Psychiatry, Psychology and Neuroscience, King's College London, London, United Kingdom; d Department of Forensic and Neurodevelopmental Sciences, Sackler Institute for Translational Neurodevelopment, Institute of Psychiatry, Psychology and Neuroscience, King's College London, London, United Kingdom; e MRC Centre for Neurodevelopmental Disorders, King's College London, London, United Kingdom; f Behavioural and Brain Sciences Unit, Population Policy Practice Programme, Great Ormond Street Institute of Child Health, University College London, London, United Kingdom; g Department of Clinical Genetics, Guy's and St. Thomas' NHS Foundation Trust, London, United Kingdom; h Program in Neurogenetics, Department of Neurology, David Geffen School of Medicine, University of California, Los Angeles, California; i Department of Human Genetics, University of California, Los Angeles, Los Angeles, California

**Keywords:** Autism, Cortical differentiation, Functional genomics, Midbrain differentiation, Neural precursors, Neural progenitor cells, Neurodevelopment

## Abstract

**Background:**

Autism is a heterogeneous collection of disorders with a complex molecular underpinning. Evidence from postmortem brain studies have indicated that early prenatal development may be altered in autism. Induced pluripotent stem cells (iPSCs) generated from individuals with autism with macrocephaly also indicate prenatal development as a critical period for this condition. But little is known about early altered cellular events during prenatal stages in autism.

**Methods:**

iPSCs were generated from 9 unrelated individuals with autism without macrocephaly and with heterogeneous genetic backgrounds, and 6 typically developing control individuals. iPSCs were differentiated toward either cortical or midbrain fates. Gene expression and high throughput cellular phenotyping was used to characterize iPSCs at different stages of differentiation.

**Results:**

A subset of autism-iPSC cortical neurons were RNA-sequenced to reveal autism-specific signatures similar to postmortem brain studies, indicating a potential common biological mechanism. Autism-iPSCs differentiated toward a cortical fate displayed impairments in the ability to self-form into neural rosettes. In addition, autism-iPSCs demonstrated significant differences in rate of cell type assignment of cortical precursors and dorsal and ventral forebrain precursors. These cellular phenotypes occurred in the absence of alterations in cell proliferation during cortical differentiation, differing from previous studies. Acquisition of cell fate during midbrain differentiation was not different between control- and autism-iPSCs.

**Conclusions:**

Taken together, our data indicate that autism-iPSCs diverge from control-iPSCs at a cellular level during early stage of neurodevelopment. This suggests that unique developmental differences associated with autism may be established at early prenatal stages.

SEE COMMENTARY ON PAGE 419

Autism spectrum conditions (henceforth referred to as autism) are a genetically heterogeneous spectrum of neurodevelopmental conditions (1, 2, 3). Autism is characterized by impairments in social-communicative behaviors as well as repetitive behaviors. Symptoms of autism cannot be detected until 12 to 18 months of age (4). However, there is debate surrounding the origins of autistic symptoms. It is now well recognized that genetic factors play a key role in the emergence of autism (1,2). Increasing evidence indicates that perturbation during critical periods of prenatal development may be key for the emergence of autism (5). Consistent with this idea, autism postmortem brain studies have identified dysregulation of putative prenatal gene expression pathways (6). Thus, early prenatal development may be a critical period for the emergence of cellular pathophysiology associated with autism (6).

The use of induced pluripotent stem cells (iPSCs) has made it possible to study prenatal cellular behavior in autism (7, 8, 9, 10, 11). iPSC neurons contain the same genetic information as the individuals from whom they were derived, and this information influences cellular behavior. iPSCs generated from individuals with autism, comorbid for macrocephaly, show significant cellular/molecular anomalies during prenatal-equivalent periods (12, 13, 14). These iPSCs demonstrated atypical neural differentiation when fated toward a cortical lineage and an imbalance in excitatory and inhibitory receptor activity (12,13). Using the same collection of iPSCs, an acceleration in neuronal maturation was found to be dependent on early cortical neural precursor development, and circumventing this stage did not recapitulate altered neuronal development. Alterations in gene expression network dynamics during early neurodevelopment also accompanied these effects (14). These studies highlight that the cellular and molecular phenotypes associated with autism may start during prenatal brain development. A critical aspect of these studies is that atypical neural differentiation was associated with higher cell proliferation (12, 13, 14). However, as the autistic participants in these studies also had macrocephaly, it is unclear whether the observed abnormal development was in part due to this comorbidity. Moreover, as macrocephaly is present only in a subset of individuals with autism, it is not known whether atypical development can be generalized to autistic individuals without macrocephaly. Finally, as most studies have predominantly focused on the development of forebrain/cortical neurons, it is yet to be tested whether atypical development can also be observed in cortical neural precursors fated toward a different lineage.

In this study, we generated iPSCs from autistic individuals without macrocephaly from 3 independent participant cohorts to capture a wider population of individuals with autism. Initial RNA-sequencing studies indicated that early neurodevelopment may be affected. To further investigate the source of atypical gene expression, we undertook extensive cellular phenotyping experiments. The goal of this study was to understand whether there was a fundamental difference between typical and autistic prenatal neurodevelopment, focusing primarily on early neuroectodermal structures and cell types that constitute the developing cerebral cortex.

## Methods and Materials

Further information can be found in Supplement 1.

### Participant Recruitment

Participants were recruited and methods carried out in accordance to the Patient iPSCs for Neurodevelopmental Disorders (PiNDs) study (NHS Research Ethics Committee No. 13/LO/1218). Informed consent was obtained before recruitment from all subjects for participation in the PiNDs study. Ethical approval for the PiNDs study was provided by the NHS Research Ethics Committee at the South London and Maudsley (SLaM) NHS Research and Development Office. Participants with autism were selected based on Autism Diagnostic Observation Schedule or Autism Diagnostic Interview–Revised scores, while typical control subjects were selected from the population if they had no diagnosis of any psychiatric condition.

All participants provided consent to report and publish their data.

### Induced Pluripotent Stem Cells

Fifteen iPSC lines (autism, 9; control, 6) were generated from hair keratinocytes as previously described (15,16). Details on all participants can be found in Supplemental Results and Tables S1 to S3 in Supplement 1. Two iPSC clones per participant were generated and used in all experiments. Pluripotency of all iPSCs was determined by immunocytochemistry and PluriTest analysis of Illumina HT12v4 transcriptome array data (https://www.pluritest.org); and genome integrity was assessed by an Illumina Human CytoSNP-12v2.1 beadchip array and analysed using KaryoStudio software (Illumina, San Diego, CA) (Table S4 and Figure S1 in Supplement 1).

### Neuronal Differentiation

iPSCs were differentiated to cortical neurons using a dual SMAD inhibition protocol that recapitulates key hallmarks of corticogenesis (10,16). iPSCs were differentiated to midbrain floorplate precursors using established protocols (7,8).

### RNA-Sequencing

RNA-sequencing was performed from a subset of our cohort, using 2 clones from each participant (ASDM1, 004ASM, 010ASM, CTRM1, CTRM2, CTRM3), with 2 technical replicates per clone. Poly(A) containing messenger RNA was purified and libraries were prepared using TruSeq Stranded mRNA kit (Illumina). Unstranded libraries with a mean fragment size of 150 base pairs were constructed and underwent 50–base pair single-ended sequencing on an Illumina HiSeq 2500 machine. Bioinformatics analysis was performed using C++-based (Standard C++ Foundation; https://isocpp.org/) and R-based (R Foundation, Vienna, Austria) programs.

### Immunocytochemistry

Differentiated iPSCs were fixed in 4% paraformaldehyde and processed as previously described (16). Briefly, fixed cells were permeabilized in 0.1% Triton-X-100/phosphate-buffered saline, and blocked in 4% normal goat serum in phosphate-buffered saline. Primary antibodies (Table S5 in Supplement 1) were incubated overnight at 4°C. Nuclei were identified by staining with DAPI. High content screening (HCS) was performed using an Opera Phenix High-Content Screening System (PerkinElmer, Waltham, MA). Immunofluorescence was measured from known intracellular location of markers (e.g., nucleus or cytoplasm). Cell type analysis was performed using the Harmony High Content Imaging and Analysis Software (16). For rate of cell-type assignment (deltaCTA or dCTA), the percentage of positively stained cells appearing per day was estimated, which was then adjusted to the total number of positive cells appearing per day in 1 well of a 96-well plate, assuming each well had an average of 10^5^ cells.

### Statistics

Quantification of cell types was performed using the Harmony High Content Imaging and Analysis Software. Percentage of cells positive for desired marker versus total number of live cells was calculated. Eight independent experimental replicates of 2 clones per individual participant were used at every stage to account for variability associated with iPSC differentiation. Independent 2-group *t* test was used to check significant difference between autism and control using *p* ≤ .05. One-way analysis of variance was performed to investigate in-group variance. All statistical analysis was performed on R statistical software.

## Results

### Neurodevelopmental Gene Expression Signatures in Autism-iPSC–Derived Neurons

**Figure 1:**
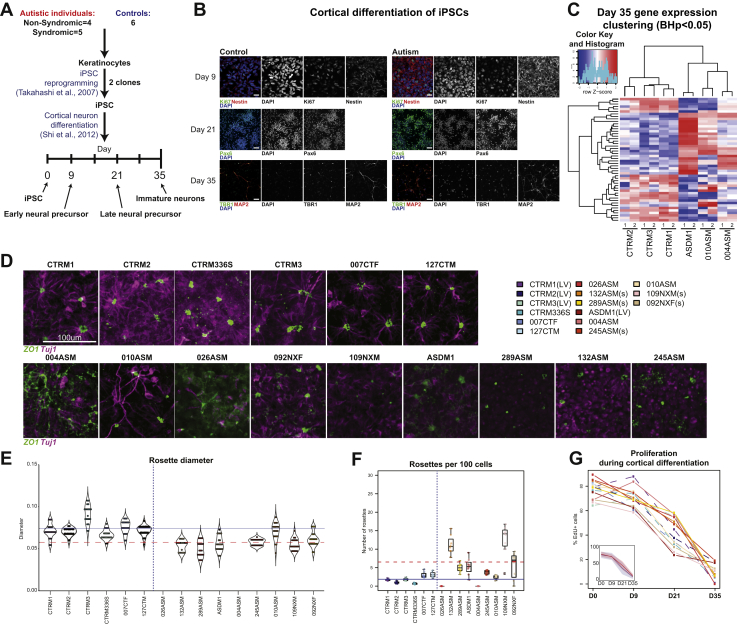
Differentiation of iPSCs into cortical lineage reveals gene expression and neural rosette formation differences between control and autism. **(A)** Study design and differentiation time points used in this study. **(B)** Differentiation of control- and autism-iPSCs generate precursor markers Ki67, Nestin, and PAX6 and neuronal markers TBR1 and MAP2. **(C)** Differential gene expression and hierarchical clustering reveals significant differences between control and autism samples (biological replicates for each sample labeled 1 and 2). **(D)** Day 9 neural rosette morphology from all participants in this study. **(E)** Rosette diameter violin plot (horizontal lines show mean rosette diameter; blue, control: red-dashed, autism). **(F)** Number of rosettes per 100 cells (horizontal lines show mean rosette number; blue, control; red-dashed, autism). **(G)** Proliferation during cortical differentiation at days 0, 9, 21, and 35 (dashed lines are control samples; color key on top right corner). BH, Benjamini-Hochberg; EdU, 5-ethynyl-2′-deoxyuridine; iPSC, induced pluripotent stem cells; LV, lentivirus reprogramming method used for generating these iPSCs; s, participants with syndromic autism; ZO-1, zonula occludens-1.

iPSC cells were generated from 9 individuals with autism and 6 typical control individuals from 3 independent cohorts (Supplemental Results and Tables S1–S3 in Supplement 1). To understand whether iPSCs derived from individuals diagnosed with autism but without macrocephaly also displayed atypical cortical differentiation with altered cell proliferation as previously reported (12,13,17), cells were differentiated toward a cortical fate. We focused on 3 distinct developmental stages (Figure 1A): 1) day 9, early neural precursor stage, when stem cells form new precursor cells that self-organize into neural tube-like structures known as neural rosettes with a directional apical-basal arrangement; 2) day 21, late neural precursor stage, a period during which neural progenitor cells begin forming layers from the apical surface and are primed for differentiation into neurons as they move outward; and 3) day 35, immature cortical neurons, a stage at which precursors become postmitotic and adopt a deep layer neuronal identity (Figure 1B). We initially sought to confirm whether day 35 neurons from autism-iPSCs showed a similar transcriptomic profile as that seen in postmortem brain studies (6,9,18). For this analysis, we chose participants with no familial history of autism or known deletions in autism-associated genes to reduce genetic bias. Analysis of differential gene expression (Figure 1C and Table S6 in Supplement 2) confirmed distinct transcriptomic profiles of control- and autism-iPSCs, and a high enrichment for genes identified in autism postmortem brain studies, but not schizophrenia or cancer (see Supplemental Results). These data suggested that differences between autism- and control-iPSCs may appear during early neurodevelopment.

### Marked Alteration in Rosette Structures in Autism Without Proliferative Differences in Precursor Pools

Differentiation of iPSCs toward a neuronal fate first results in the generation of neuroepithelium cells, which self-organize into structures known as “neural rosettes” (10). These structures display apical-basal polarity similar to that of neural tubes (10,19). They are thought to play a key role in determining cortical neurogenesis and thus generation of distinct cell fates (10,19,20). As our RNA-sequencing data indicated that early neurodevelopment may be affected in autism, we reasoned that this may be reflected by an alteration in neural rosette formation. We examined rosette formation at day 9 in control- and autism-iPSCs. Control-iPSCs robustly formed structures identifiable as neural rosettes, with an inner lumen identified by ZO-1 (zonula occludens-1) staining. Neural progenitor cells self-organized radially around the inner lumen, typical of cells adopting an apical-basal polarity organization (Figure 1D). Conversely, autism-iPSCs showed significant anomalies in lumen formation and establishment of apical-basal polarity (Figure 1D). Using an HCS approach, we assessed rosette structure to identify consistent alterations in rosette morphology between iPSC lines. All 6 control-iPSC lines formed rosettes similar in structure (average diameter, 0.066–0.091 mm) (Figure 1E and Table S7 in Supplement 1). Conversely, of the 9 autism-iPSCs, 6 iPSC-lines formed rosettes with a smaller diameter (0.05–0.06 mm); 2 did not form any rosette structures at all (026ASM and 004ASM; both clones); while 010ASM formed rosettes with diameters similar to those of control-iPSC lines (0.07 mm) (Figure 1E and Table S7 in Supplement 1). Autism-iPSC lines also formed more rosettes per 100 cells (Figure 1F and Table S7 in Supplement 1). Anomalous formation of rosettes was recapitulated in day 30 3-dimensional cortical spheroids (Figure S4A in Supplement 1), with fewer complete rosettes observed in autism-iPSC spheroids (Figure S4B in Supplement 1). One explanation for these observed morphological differences could be that autism-iPSCs have altered levels of cell proliferation. However, all control- and autism-iPSCs had similar rates of cell proliferation at each developmental stage examined (Figure 1G). Together, these data show that autism-iPSCs form anomalous rosettes independent of alterations in cell proliferation.

### Divergence From Typical Development in Autism Occurs at Precursor Cell Stages During Cortical Differentiation

**Figure 2:**
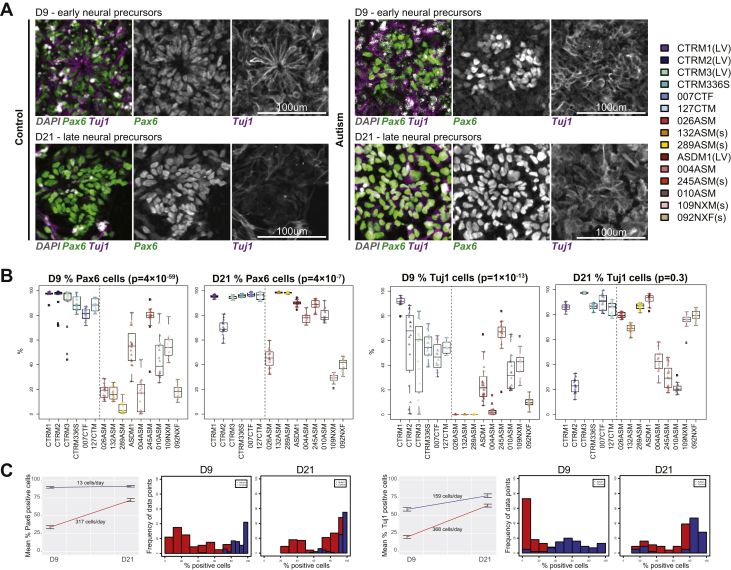
Atypical cortical differentiation of autism induced pluripotent stem cells. **(A)** At day 9 and day 21 precursor cell stages, both control– and autism–induced pluripotent stem cells expressed PAX6 and Tuj1. **(B)** Quantification of PAX6+ and Tuj1+ cells of individual participants (percentage of cells positive per experimental replicate) showed significant differences between the autism and control samples. **(C)** Mean values of percentage of positive cells over time show significant difference between control and autism groups at days 9 and 21, as well as significant difference in rate of appearance of markers. Histogram shows normal distribution of experimental data points and demonstrates variability between control and autism samples. D, day; LV, lentivirus reprogramming method used for generating these induced pluripotent stem cells; s, participants with syndromic autism.

Abnormal rosette proliferation observed in autism-iPSCs could indicate premature or atypical neuronal differentiation in autism-iPSCs. To investigate this possibility, we assayed cortically differentiating iPSCs at critical stages of cortical differentiation—days 9, 21, and 35 (Figure 1A, B)—for fundamental developmental markers using an HCS-based approach. First, we assessed the expression of PAX6 and Tuj1 in control- and autism-iPSCs (Figure 2A). PAX6 is a robust marker for neural precursors of cortical lineage (21), while Tuj1 is a robust pan-neuronal and neural precursor marker (22). Eight independent experimental replicates using 2 clones per line were assayed at every stage (Figure 2B). At day 9, PAX6 and Tuj1 were expressed in majority of control-iPSC cells (Figure 2B and Table 1). On day 21, both markers remained highly expressed (Figure 2B and Table 1). We also measured dCTA as an independent way to compare how quickly cell identity was being acquired or lost between developmental stages. In control-iPSCs, PAX6 dCTA was 13 cells/day between days 9 and 21, while for Tuj1, dCTA was 159 cells/day (Figure 2C). In contrast in the autism group, expression of PAX6 and Tuj1 at day 9 was lower than in the control group (Figure 2B and Table 1). Assessment of cell identity acquisition in autism-iPSCs showed that PAX6 dCTA was 317 cells/day and Tuj1 dCTA was 368 cells/day. These values were higher than those observed following the differentiation of control-iPSCs. However, despite this increased rate of cell identity acquisition, PAX6 and Tuj1 expression was still significantly lower at day 21 in autism-iPSCs than in control-iPSCs (Figure 2C and Table 1). As expected, variability was observed throughout the differentiation protocol between experimental replicates. However, this variability was more pronounced in the autism-iPSCs. Analysis of variance revealed greater overall spread of data points and higher *F* values in the majority of parameters assessed during differentiation of autism-iPSCs. Of note, individual clones from each line behaved in a similar manner, indicating that the use of multiple clones was not the source of variability (Figure S5A, C and Table S8 in Supplement 1). Moreover, nonsyndromic and syndromic samples appeared to behave similarly (Figure S6A, C, and D in Supplement 1). These data showed that control-iPSC–derived precursors expressed PAX6 and Tuj1 early during differentiation, while autism-iPSCs displayed lower PAX6 and Tuj1 expression at the equivalent stage. Beyond this stage, the rate of acquisition of PAX6 and Tuj1 was higher in autism-iPSCs, and the difference between control- and autism-iPSCs was substantially reduced at day 21.

**Table 1:** Percentage of Cells Expressing Neural Differentiation Markers

Marker	Day 8—Early Precursors (%)			Day 21—Late Precursors (%)			Day 35—Neural Cells (%)		
	Control	Autism	*p* Value	Control	Autism	*p* Value	Control	Autism	*p* Value
PAX6	93.54545	33.88251	4 × 10^−59^	86.66410	71.94075	4 ×10^−7^	–	–	–
Tuj1	65.17584	19.87218	1 × 10^−13^	68.68563	64.00949	.3^a^	–	–	–
EMX1	95.69082	79.65836	4 × 10^−11^	88.5446	80.8861	.003	65.83102	50.35212	.01
GAD67	33.223989	4.406441	1 × 10^−8^	28.04423	26.66252	.55^a^	20.05228	47.78413	3 × 10^−9^
TBR1	–	–	–	–	–	–	59.07799	50.07018	.03

Independent 2-group *t* test was performed between control and autism values for each time point (*p* ≤ .05). PAX6 and Tuj1 expression at day 35 was not observed as there are 0 PAX6 cells in terminally differentiated neurons, while all terminally differentiated cells of neuronal lineage express Tuj1.

a Not significant.

### Altered Development of Forebrain Precursor Lineages in Autism-iPSCs Independent of Cell Proliferation

**Figure 3:**
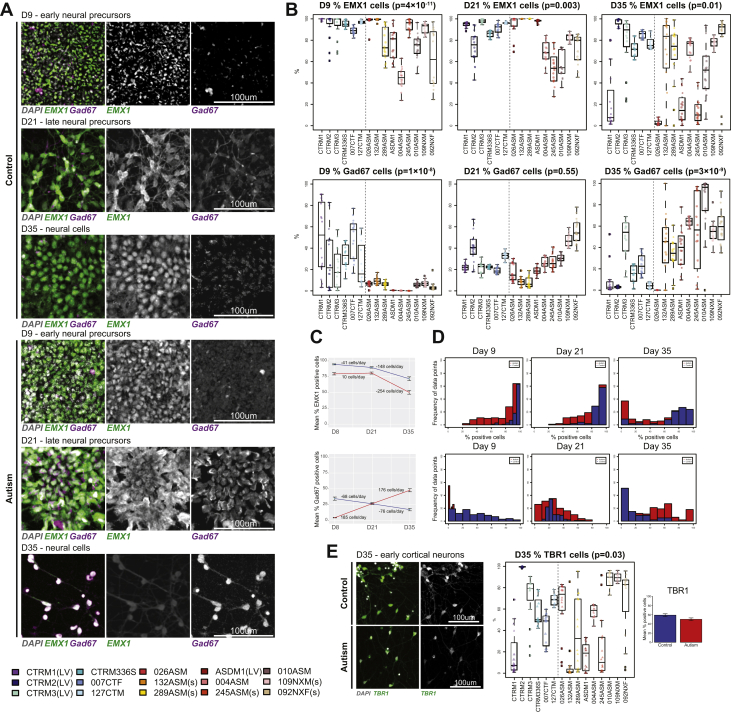
Atypical differentiation into dorsal and ventral forebrain precursors in autism. **(A)** EMX1 was expressed at day 9, day 21, and day 35 in both control and autism groups. GAD67 expression in both groups was time dependent, it decreased over time in in the control sample, while it increased over time in the autism sample. **(B)** Quantification of EMX1+ and GAD67+ cells (percentage of cells positive per experimental replicate) showed significant differences between autism and control samples. **(C)** Mean values of percentage of positive cells over time show significant difference between control and autism samples at every time point, except for GAD67 at day 21 of the precursor stage. **(D)** Histogram shows normal distribution of experimental data points and clear difference in distribution of data points between groups. **(E)** Control– and autism–induced pluripotent stem cells also expressed TBR1 at day 35 of cortical differentiation, and TBR1 expression was marginally higher in control vs. autism samples. D, day; LV, lentivirus reprogramming method used for generating these induced pluripotent stem cells; s, participants with syndromic autism.

Previous iPSC studies have linked an imbalance in GABA (gamma-aminobutyric acid)–glutamatergic progenitor cells and neuronal function with a macrocephaly-associated cell proliferation phenotype (13,17). Thus, we were interested in establishing whether a similar imbalance in the presence of GABA-glutamatergic progenitor cells could be observed in our autism-iPSCs. We investigated the development of precursors expressing EMX1, known to be expressed in dorsal forebrain (glutamatergic) neurons and precursors (23, 24, 25), and GAD67, the rate-limiting enzyme in the GABA synthesis pathway and known to be expressed in GABAergic cells (26,27) (Figure 3A). At day 9, EMX1 expression was significantly higher in control compared with autism neural precursors (Figure 3B, C and Table 1). At day 21, EMX1 expression in both groups appeared to remain stable, with only minor reduction in control precursors (dCTA = −41 cells/day), as opposed to a minor increase (dCTA = +10 cells/day) in the autism group (Figure 3C). At this stage, control neural progenitors expressed EMX1 significantly higher than autism neural progenitors (Figure 3B, C and Table 1). In day-35 immature neurons, EMX1 expression in both control and autism neurons was reduced compared with the expression in day-9 and -21 precursors; however, the reduction was significantly more acute in the autism group (dCTA = −148 cells/day in control-iPSCs vs. dCTA = −254 cells/day in autism-iPSCs) (Figure 3C). GAD67 expression in autism- and control-iPSCs followed an opposing trajectory. At day 9, GAD67 expression was significantly higher in the control precursors, while autism precursors displayed negligible expression (Figure 3B, C and Table 1). At day 21, GAD67 expression was reduced in the control progenitors (dCTA = −68 cells/day) but was significantly increased in autism neural progenitors (dCTA = +185 cells/day) (Figure 3C). Control and autism progenitors had similar GAD67 expression at this stage (Figure 3B, C). However, by day 35, GAD67 expression in autism neurons was higher than that in control neurons (control dCTA = −76 cells/day, autism dCTA = +176 cells/day) (Figure 3C, D). Similar to what we observed with PAX6- and Tuj1-expressing cells, EMX1 and GAD67 expression also showed conspicuous variability. Again, analysis of variance revealed greater variability in majority of the parameters in autism lines, with no contribution of clones to the observed variability (Figure S5B, C and Table S8 in Supplement 1). Nonsyndromic and syndromic samples behaved in a similar manner (Figure S6B–D in Supplement 1). Lastly, we examined the expression of TBR1, a transcription factor expressed in early born excitatory neurons (10,28), in day-35 neurons. This revealed that differentiated control-iPSCs had higher levels of TBR1-positive cells than differentiated autism-iPSCs (Figure 3E). Taken together, these data showed significant differences in the determination of neuronal subtype identity of cortical lineage, in control- and autism-iPSCs.

### Generation of Midbrain Floorplate Progenitors Reveal Negligible Differences Between Control- and Autism-iPSCs

**Figure 4:**
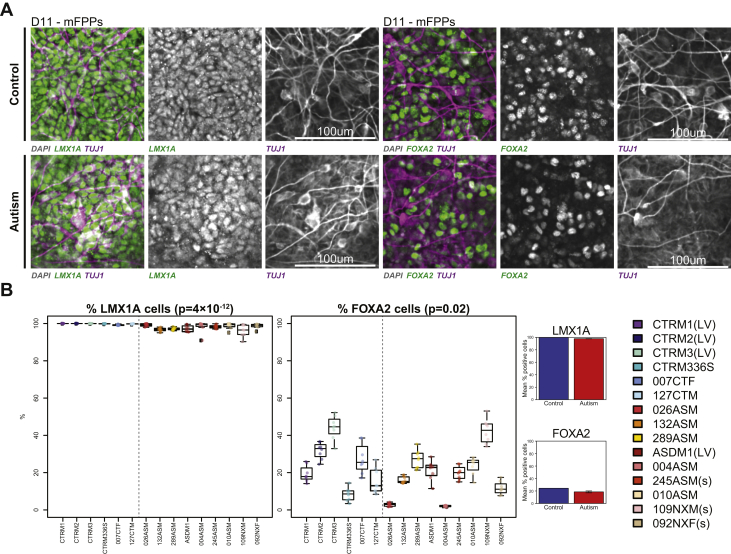
Efficient differentiation of control and autism-iPSCs toward a midbrain fate. **(A)** Both control- and autism-iPSCs expressed LMX1A and FOXA2 when differentiated into mFPP cells. **(B)** Differences between control and autism-iPSCs expressing LMX1A or FOXA2 was near negligible. iPSC, induced pluripotent stem cells; LV, lentivirus reprogramming method used for generating these iPSCs; mFPP, midbrain floor plate precursor; s, participants with syndromic autism.

The differences in cell fate acquisition observed between control- and autism-iPSCs could be due to genetic differences between control- and autism-iPSCs. Alternatively, this variation could be due to stochastic fluctuations in activation of key transcription factors during differentiation, as reported during iPSC differentiation toward a cortical fate (29). However, these differences could also be due to an inherent abnormality in the ability of our study’s autism-iPSCs to undergo neural differentiation. Therefore, we sought to determine whether both control- and autism-iPSCs differentiated efficiently into neural progenitor cells specific for another neuronal linage, specifically, mesencephalic dopamine neurons. We chose this fate as mesencephalic dopamine neurons are generated from midbrain floor plate progenitors (mFPPs) that arise from cells located on the ventral midline of the neural tube floor plate. The generation of mFPPs would, therefore, require a distinct set of factors compared with those needed for the generation of cortical precursor cells. While dysfunction in mesencephalic dopamine neurons has been linked with Parkinson’s disease and schizophrenia, there are fewer reports of dysfunction in these cells in autism. Therefore, we reasoned that generating mFPPs (7,8) allowed us to examine the differentiation capacity of our iPSCs. After 10 days of differentiation, nearly 100% of mFPPs from both control- and autism-iPSCs were positive for LMX1A, an essential transcription factor required for defining a midbrain identity (30) (Figure 4A, B). No difference was observed between control- and autism-iPSCs. Similarly, expression of the transcription factor FOXA2, which positively regulates neurogenic factors in dopaminergic precursor cells (31), did not differ between control and autism mFPPs (Figure 4A, B). Variability was also reduced in all the iPSC lines during midbrain differentiation (Figure 4B). Taken together, these data showed considerably reduced differences in midbrain lineage differentiation between control- and autism-iPSCs.

### Hierarchical Clustering Reveals Subgrouping of Study Participants Based on Cellular Phenotypes Alone

**Figure 5:**
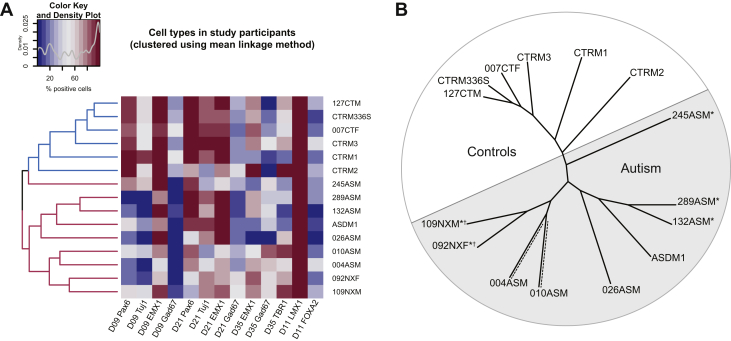
Hierarchical clustering of cellular data using mean linkage method. **(A)** All control and autism-iPSC lines were grouped based on percentage of positive values for PAX6, Tuj1, EMX1, and GAD67 at day 9, day 21, and day 35 cortical differentiation and LMX1A and FOXA2 at day 11 of midbrain differentiation. Control participants and participants with autism were grouped separately using this unsupervised learning method. **(B)** Unrooted phylogenetic tree showing relatedness of individual participants based on cellular phenotypes. ∗Syndromic samples branched separately to nonsyndromic samples. ∗^†^NRXN1 deletion samples grouped together on the same branch. 004ASM and 010ASM, which grouped on the same branch (shown with dashed lines), also grouped similarly based on gene expression data shown in Figure 1C and Figure S3B in Supplement 1. D, day; iPSC, induced pluripotent stem cell.

The findings in this study indicate that there may be a link between autism and prenatal cortical development. Our HCS approach has allowed us to collect large data sets of multiple cellular readouts at a number of developmental time points for each iPSC line. Hierarchical clustering approaches allow for the identification of similar patterns between samples by placing them into cluster sets (32). Using this approach, we tested whether there was a relationship between atypical cortical neurogenesis and diagnosis, based on cellular phenotypes from control- and autism-iPSCs. Data points from each iPSC line were amalgamated into a heat map (Figure 5A), and participants were ordered on the heat map based on a mean linkage method. We then visualized the clustering in the form of an unrooted dendrogram (Figure 5B), as participants in this study were unrelated (33). We discovered notable relationships between samples. First, the control participants and participants with autism were grouped separately. Within the autism cluster, the participants with *NRXN1* deletions (109NXM and 092NXF) were grouped on the same branch. Three participants with syndromic autism (109NXM, 092NXM, 245ASM) did not group together with the nonsyndromic participants. Lastly, the 2 autism samples 004ASM and 010ASM seemed to group on the same branch based on not only the cellular data points but also gene expression patterns in Figures 1C and Figure S3B in Supplement 1. The individual patterns that emerged out of this unbiased analysis suggest that there is a potential that cellular phenotypes could reflect the nature of autism diagnosis. Further studies using larger collections of deeply phenotyped iPSCs as well as more detailed cellular readouts are needed to understand whether such an association is robust over independent cohorts.

## Discussion

In this study, we investigated whether iPSCs generated from autistic individuals display differences during prenatal cortical development. Previous studies have indicated that prenatal development is a critical period for the emergence of phenotypes associated with autism (12, 13, 14). However, these studies utilized iPSCs generated from individuals with autism comorbid with macrocephaly, making it unclear whether the observed cellular effects were due to autism or altered brain size. We studied iPSCs generated from a heterogeneous group of autistic individuals without macrocephaly, recruited from 3 independent cohorts, and were able to test whether altered cellular identities occurred during differentiation of autism-iPSCs toward cortical fate and whether this was detectable from an early developmental stage. This collection included 4 autistic individuals with uncharacterized genetic background and 5 autistic individuals with copy number variations in high-risk autism loci.

First we found that autism-iPSCs generated atypical neural rosettes, indicating an alteration in neural differentiation. Consistent with this, autism-iPSCs showed significant differences in development of early neural progenitor cells. This effect persisted at a late precursor cell stage, although to a lesser degree. No differences in proliferative capacities were observed between control- and autism-iPSCs, indicating that this was not the cause of altered neurogenesis in autism-iPSCs. Examination of cortical neuron subtypes revealed a divergence in the development of dorsal forebrain or excitatory precursors and ventral forebrain or inhibitory precursors from an early stage of development. Conversely, control- and autism-iPSCs demonstrated the same ability to differentiate into mFFP cells. This indicates that atypical neurogenesis predominantly impacts the development of cortical lineages in autism-iPSCs. Finally, based on all the temporal cortical data points acquired in this study, the participants were grouped separately into control and autism groups, with further unbiased branching within the autism cohort. Together, these data suggest that unique developmental differences associated with autism may be established at early prenatal stages.

We were particularly interested in modeling divergent patterns of development in the autistic cortex. We used a cortical differentiation protocol that recapitulates cortical precursor development from iPSCs and that yielded primarily excitatory cortical neurons (10). This enabled us to study early stages of neural development, when neural rosette begins forming (day 9), equivalent to neural tube closure (approximately 4 weeks of gestation) (34). We found marked anomalies in rosette morphology in 3 of 9 autism-iPSCs (004ASM, 026ASM, 245ASM), resulting in either malformation or negligible neural rosette formation. In 010ASM, neural precursors were visibly dissociated from the rosette structure, while in 092NXF, 109NXM, ASDM1, 132ASM, and 289ASM, cells appeared elongated and lumen formation was also affected. Further studies are required to elucidate the mechanisms responsible for the altered rosette structures and formation observed. Disruption of neural rosettes has been found to promote premature neurogenesis (35,36). This may explain the high rate of PAX6+ and Tuj1+ precursor generation between days 9 and 21 in autism-iPSCs. It could also explain divergent precursor subtype assignment during early development, which we observed through opposing trajectory of GAD67-expressing cells in control- and autism-iPSCs. We noted that the appearance of GAD67+ cells in our cultures was surprising as SMAD inhibition is known to drive stem cells toward a dorsal forebrain lineage, while GABAergic neurons are known to be generated from a ventral forebrain lineage (37). However, low numbers of GABAergic cells are known to be generated using the SMAD inhibition protocol (38,39), and appearance of GAD67+ cells and their dysregulation in our study may be a result of dysregulated molecular mechanisms associated with atypical precursor subtype assignment.

It is of interest that in the current study the phenotypic changes occurred without the presence of proliferative differences between control- and autism-iPSCs. This suggested that cell type and structural anomalies previously reported using autism-iPSCs (12,13) may be independent of macrocephaly-associated cell proliferation alterations. Alterations in rosette formation may also contribute to the switching of precursor identity seen during development in autism-iPSCs. Further investigation into temporal precursor cell type specification will be needed to understand the mechanisms and types of cells involved. Notably, iPSC studies of nonsyndromic autism remain underpowered. Nevertheless, the reports of neurodevelopmental differences between autism- and control-iPSCs are robust (13,14,40). Although our cohort size would be considered inadequate for a study into nonsyndromic autism, it is comparable to recent iPSC-based psychiatric studies (12, 13, 14,41). To achieve effect size in our study, we have used multiple clones for each iPSC line. In addition, we utilized an HCS approach of “cellomic” cell-based phenotyping (15,16,42), recording thousands of data points from each iPSC line.

Another consideration we faced during cellular phenotyping of iPSCs being differentiated toward a cortical fate was the high degree of variability between experimental replicates. This variability is due in part to stochastic fluctuations in transcription factor activation during cortical differentiation (29,43). We observed that of the 10 temporal data points recorded, 7 showed a greater degree of variability in autism-iPSCs. To rule out whether this variability was due to an iPSC-related abnormal artifact, we differentiated both control- and autism-iPSCs toward a mesencephalic fate. Following this protocol, iPSCs from either control individuals or individuals with autism behaved similarly and demonstrated reduced variability. This suggests that the variability observed in this study is specific to cortical differentiation rather than an iPSC-related artifact. Moreover, these data indicate that alteration during an early stage of development associated with autism may occur in a region-specific manner.

In this study, we used iPSCs generated from independent cohorts and from individuals with autism but without macrocephaly. Using unbiased methods, we identified that differentiation of autism-iPSCs toward a cortical, but not a mesencephalic, fate results in atypical neurogenesis characterized by premature maturation and abnormal specification of neural progenitor cells. These effects occur in the absence of altered proliferative activity between control- and autism-iPSCs. Identification of these cellular/molecular phenotypes enabled us to find common cellular pathways in a cohort having heterogeneous genetic background. In future, similarly designed studies will help identify which cellular pathways underlie these phenotypes and may help to improve diagnosis and develop a greater understanding of the origins of autism.

## Supplementary Material

Supplement 2

Key Resources Table
