# Supplementary material for: Atypical Neurogenesis in Induced Pluripotent Stem Cells From Autistic Individuals
Source: Biol Psychiatry. 2021 Mar 1;89(5):486–96. doi: 10.1016/j.biopsych.2020.06.014 (PMC7843956; doi:10.1016/j.biopsych.2020.06.014)
Supplement: Supplement 1 [file mmc1.pdf]

Atypical Neurogenesis in Induced Pluripotent Stem Cells From Autistic Individuals

Supplement 1

Supplement Contents

Extended Experimental Procedures

Supplementary Results

Supplementary Figure S1

Supplementary Figure S2

Supplementary Figure S3

Supplementary Figure S4

Supplementary Figure S5

Supplementary Figure S6

Supplementary Table S1

Supplementary Table S2

Supplementary Table S3

Supplementary Table S4

Supplementary Table S5

Supplementary Table S7

Supplementary Table S8

Supplementary References

Extended Experimental Procedures

Study participants and neuronal differentiation

Keratinocytes were collected from autistic participants, and typical controls without an autism diagnosis (Ethics approved, 13/LO/1218) as part of a larger European studies (EU-AIMS, STEMBANCC). All participants were Caucasian, while controls were selected if they did not have diagnosis of any psychiatric conditions. These were reprogrammed into iPSCs using previously described methods (1, 2). IPS cells were cultured in E8 medium (Life Technologies) with E8 supplement (Life Technologies). Cell type quantification and proliferation assays were set up on 96-well plates. 2 clones were selected from each participant, and each clone had 8 technical replicates. RNA-sequencing was performed on 2 clones from each participant, and each clone had 2 technical replicates. This design was maintained at all stages of neural differentiation recorded. Induction of neurons of cortical lineage was established using a modified dual SMADi protocol (3). Once the cell culture reached 95% confluence, neural induction was initiated by changing the culture medium to support neural induction, neurogenesis and neuronal differentiation. A combination of N2- and B27-containing media with additives was used, henceforth called 'neuralising medium'. N2 medium consisted of DMEM/F12 (Sigma), N2 (Gibco). B27 medium consisted of Neurobasal (Invitrogen), B27 (Gibco). Neuralising medium was supplemented with 'dual SMADi' 1 μM Dorsomorphin (Tocris), 500 ng/ml human Noggin-CF chimera (R&D Systems) – inhibitors of WNT pathway, BMPs and SMAD, and 10 μM SB431542 (Tocris) – inhibitor of TGFβ signaling. Noggin and dorsomorphin supresses embryonic development thereby inducing neural differentiation pathways, while SB431542 mediates loss of pluripotency. Midbrain floorplate precursors were differentiated from all participants till day 11 using previously established protocols (4, 5). To generate cortical spheroids, iPSCs were first treated with

KOSR-based hiPSC media to form embryoid bodies. Neural induction was performed using dorsomorphin and SB431542. After 6 days of neural induction, spheroids were transferred into neural maintenance media till day 30, as per established protocols (6).

Cortical spheroids

Cortical spheroids were generated using methods published by Pasca *et al.* (2015). Human iPSCs were passaged at high density (80 % confluent) as previously described. The cells were then suspended using a cell lifter in KOSR media, 10 μM ROCKi and SMAD inhibitors: 5 μM Dorsomorphin and 10 μM SB431542. The cultures were incubated in a 5% CO2 incubator and left undisturbed for 48-hours period to promote formation of embryoid bodies. Starting the second day and until the fourth day media is changed daily with fresh KOSR media supplemented with 5 μM Dorsomorphin and 10 μM SB431542. By the fifth day embryo bodies were clearly visible. At this stage, till 25th day the culture media used was B27 minus Vitamin A media plus 20 ng/mL of recombinant human EGF and recombinant human bFGF. From day 25, B27 minus Vitamin A media with 20 ng/mL of recombinant human BDNF and recombinant human NT3 was used. Media was changed every 24 hours during the first 15 days and once every 48 hours thereafter. The spheroids were harvested at day 30.

Immunocytochemistry

Cultures were fixed in 4% formaldehyde followed by ice-cold 100% methanol and processed for immunofluorescence staining, confocal microscopy and high throughput imaging. Secondary antibodies used for primary antibody detection were species-specific Alexa-dye conjugates (Invitrogen). We used the following primary antibodies to Ki67 (Thermo Fisher PA5-16785), Nestin (Millipore MAB5326), Pax6 (BioLegend 901301), TBR1 (Abcam ab31940), MAP2 (Abcam ab92434), Emx1 (ThermoFisher PA5-35373), Gad67 (Abcam ab26116), Tuj1 (BioLegend 801201), CD44 (R&D Systems MAB7045), LMX1A (Abcam

ab139726), FOXA2 (Invitrogen 701698). Quantification was performed on the Perkin Elmer Harmony Software v4.9, which is based on the CellProfiler high throughput image analysis system (7). Cell nuclei were first identified based on DAPI staining. For nuclear proteins, only the nuclear area was selected. For cytoplasmic protein, the area around the nucleus was selected. Thresholds of fluorescent intensity was selected after background subtraction. Threshold for each probe remained unchanged for every sample imaged. Antibodies, dilutions used and fluorescence threshold information in **Supplementary Table S5**.

Cortical spheroids were first washed in PBS and then fixed in 4% formaldehyde in 4% sucrose-PBS for 45 minutes. Following fixation, spheroids were washed in PBS and stored in sucrose 30% (weight/volume); sucrose sinking improves their preservation. After sucrose sinking, spheroids were permeabilised in 2% normal goat serum (NGS) in 0.1% Triton X100 in PBS for 60 minutes. Spheroids were incubated in a solution of permeabilization-blocking solution containing the primary antibodies for 48 hours (**Supplementary Table S5**). The samples were washed three times for three minutes in PBS and incubated with permeabilisation-blocking solution containing the secondary antibodies and HOECHST (nuclear staining) for two hours and kept in darkness to prevent bleaching of the fluorophores. Cortical spheroids were mounted as whole tissues (without sectioning), due to their small size (1-5 mm). Cortical spheroids were imaged using a Lecia SP5 confocal microscope using a 40x oil immersion objective. Imagines were acquired as Z stacks at resolution 1024x1024px and employing multiple channels: 405 (DAPI/HOECHST, blue), 488nm (green), 561nm (red) and 633nm (far-red). A line average of three was used in each channel during point scanning to prevent random background signal due to light scattering.

EdU labelling

IPSCs at specific stages of differentiation were labelled with EdU (5-ethynyl-2′-deoxyuridine) using the Click-iT EdU Assay (Invitrogen). Cells were incubated with EdU for 4 hours at 37°C, then an additional 4 hours with EdU-free media. After incubation, labelled cells were fixed and prepared for detection using the Click-iT reaction cocktail. Nuclei were labelled using Hoechst 33342. Number of EdU-labelled cells were recorded as a percentage over total number of live nuclei. Imaging and analysis were done using the Opera Phenix HCS and Harmony Analysis Software.

RNA isolation and sequencing

RNA from 2 technical replicates was extracted using 2 clones from each participant (total: 4 samples per participant). TRIzol (Thermo Fischer) method was used, replacing chloroform with 1-bromo-3-chloropropane (BCP; Sigma). To remove genomic DNA during processing, turbo DNase (Thermo Fischer) was used. RNA concentration was quantified using Ribogreen assay (Invitrogen).

Starting with 500ng of total RNA, poly(A) containing mRNA was purified and libraries were prepared using TruSeq Stranded mRNA kit (Illumina). Unstranded libraries were constructed and underwent 50bp single ended sequencing on an Illumina HiSeq 2500 machine. To analyse iPSC mRNA-seq data, the raw reads were mapped to the human genome GRCh37.75 (UCSC version hg19) using STAR: RNA-seq aligner (8). Aligned reads were sorted using samtools (9), while biases were removed using Picard tools (Broad Institute). Quality control was performed using Picard tools (Broad Institute) and QoRTs (10). Gene expression levels were quantified using an union exon model with HTSeq (11), which uses uniquely aligned reads. Only the genes with >10 reads and expressed in 80% of the samples,

were kept. The resulting read counts were log2 transformed and GC content, gene length, and library size normalised using the cqn package (12) in R.

mRNA weighted co-expression network analysis

Co-expression network analysis was performed using the R library, WGCNA (13). We wanted to investigate autism-specific iPSC-neuronal culture co-expressed genes (or modules). Biweighted mid-correlations were calculated for all pairs of genes, then a signed similarity matrix was created. In the signed network, the similarity between genes reflects the sign of the correlation of their expression profiles. The signed similarity matrix was then raised to power β to emphasize strong correlations on an exponential scale. The resulting matrix (known as adjacency matrix) was then transformed into a topological overlap matrix. Since we are primarily interested in exploring co-expressed genes conserved across our cohort, we created consensus networks correlated to autism as previously published (14, 15). After scaling for each individual network (consensus scaling quantile = 0.2), a soft thresholding power of 14 was chosen (as it was the smallest threshold that resulted in a scale-free R^2 fit of 0.8). The consensus network was created by using a topological overlap matrix (TOM) to calculate the component-wise minimum values for topological overlap. Using dissTOM = 1 – TOM as distance measure, genes were hierarchically clustered. Modules were then assigned using a dynamic tree-cutting algorithm (cutreeHybrid, using default parameters except deepSplit = 4, cutHeight = 0.999, minModulesize = 100, dthresh=0.1 and pamStage = FALSE).

Resulting modules of co-expressed genes were used to calculate module eigengenes (MEs; or 1st principal component of the module). MEs were correlated to biological traits, in this case autism, to find disease-specific modules. Module hubs were defined by calculating module membership (kME) values which are the Pearson correlation between each gene and corresponding ME, and genes with kME < 0.7 were removed from the module. Network

visualisation was done using iGraph package in R (16). Differentially expressed genes, and gene module assignments in **Supplementary Table S6**.

Enrichment analysis for gene sets

Two types of gene set enrichments were performed. For autism-correlated module enrichment, logistic regression was performed using already published gene modules (14, 15, 17) to control for gene length and gene expression level. A two-sided Fisher exact test with 95% confidence interval was performed for cell-type enrichment analysis using published human brain dataset (18).

Module genes were characterised using GO Elite (version 1.2.5) (19) using total expressed genes as background. GO Elite uses a Z-score approximation of hypergeometric distribution to assess term enrichment, and removes redundant GO or KEGG terms to give a concise output. 10,000 permutations were used, and required at least 10 genes to be enriched in a given pathway at a Z-score of at least 2. Only biological process and molecular function categories are reported. Pipeline schematic in **Supplementary Figure S2**.

Supplementary Results

Participant overview

Participants were recruited from the Longitudinal European Autism Project (LEAP) (20); Brain and Body Genetic Recourse Exchange (BBGRE) studies (21); or the Social Communication Disorders Clinic at Great Ormond Street Institute of Child Health (GOS-ICH). Of the autistic participants, eight were male and one was female (**Supplementary Table S1**). The four participants from the LEAP cohort were diagnosed with non-syndromic, while participants

from BBRGE and GOS-ICH cohorts were diagnosed with syndromic autism (**Supplementary Table S2**). Syndromic participants from GOS-ICI had deletions type CNVs in the 1p21.3 and 8q21.12 regions, with *DYPD* and *PTBP2* and the *AXL* genes of note in each region respectively. Of the syndromic participants from BBGRE, two syndromic participants had deletion type CNVs in the 2p16.3 region (*NRXN1*), while the third carried a duplication in the 3p chromosomal region (22) (**Supplementary Table S3**).

Transcriptomic analysis of iPSCs reveal enrichment of gene modules associated with autism

As *post-mortem* brain studies of adult brains have identified prenatal gene expression pathways as being altered in autism (14, 15, 17), we were interested in determining if we could observe similar altered gene expression networks in our cohort of iPSCs. Day 35 neurons were generated from three control and three non-syndromic autism-iPSCs. We chose participants with no familial history of autism or known deletions in autism-associated genes to reduce genetic bias that could drive atypical gene expression. Using an adapted bioinformatics pipeline (14, 17), we analysed gene expression pathways and assess its relatedness to autism (**Supplementary Figure S2**). Principle component analysis revealed distinct separation between the control- and autism-iPSC neurons (**Supplementary Figure S3A**). Differential gene expression (DEG) and hierarchical clustering grouped control- and autism-iPSC neurons on different branches (**Supplementary Figure S3B, Figure 1C; Supplementary Table S6**). Weighted gene co-expression analysis (WGCNA) revealed 11 gene modules significantly altered in autism-iPSC neurons (**Supplementary Figure S3C; Supplementary Table S6**). The three most upregulated and three most downregulated gene modules were strongly enriched respectively in autism *post-mortem* brain gene modules (**Supplementary Figure S3D**). These gene modules showed little to no enrichment in schizophrenia or cancer gene modules (**Supplementary Figure S3E**) indicating that the gene expression patterns in our

samples were autism-specific. From this we concluded that altered gene expression in adult autism brains was also found in prenatal neurons generated from iPSCs, and that gene expression patterns were specific to autism.

Supplementary Figures

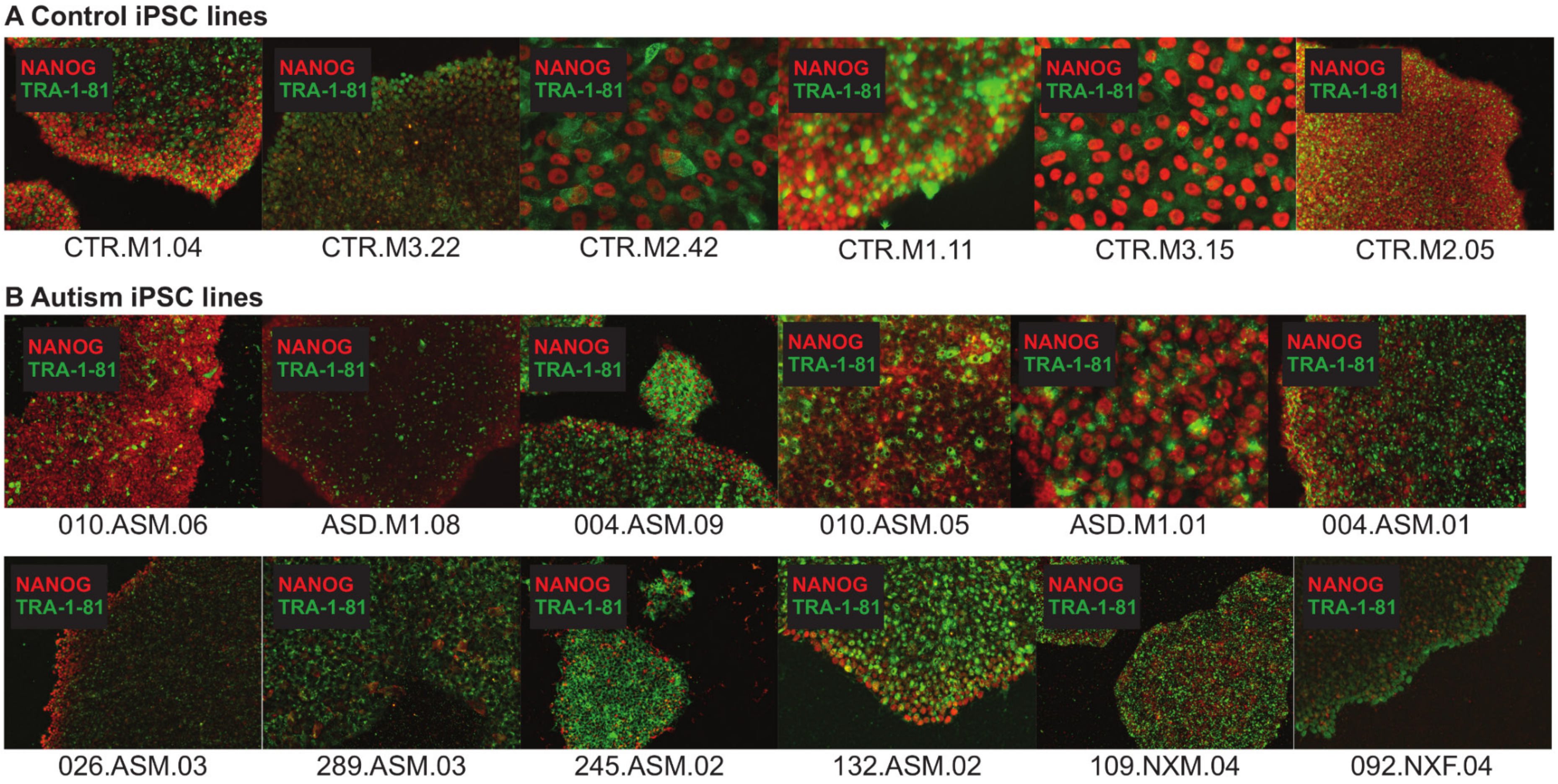

Supplementary Figure S1: Quality control images of iPSC lines. Pluripotency of all iPSC lines were determined by positive staining for stem cell markers NANOG and TRA-1-81.

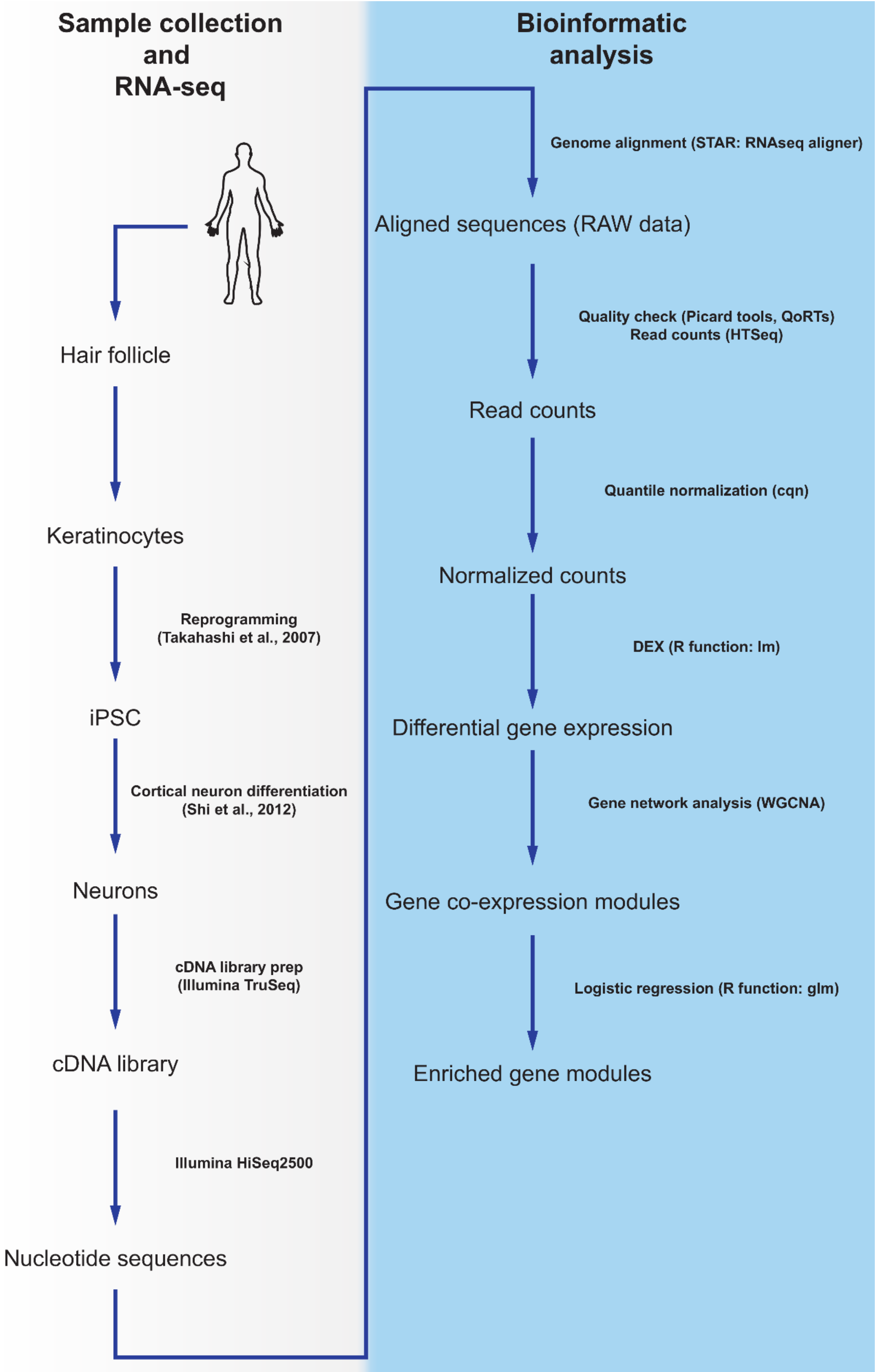

Supplementary Figure S2: Bioinformatics pipeline. Analysis pipeline for RNASeq.

Figure S3

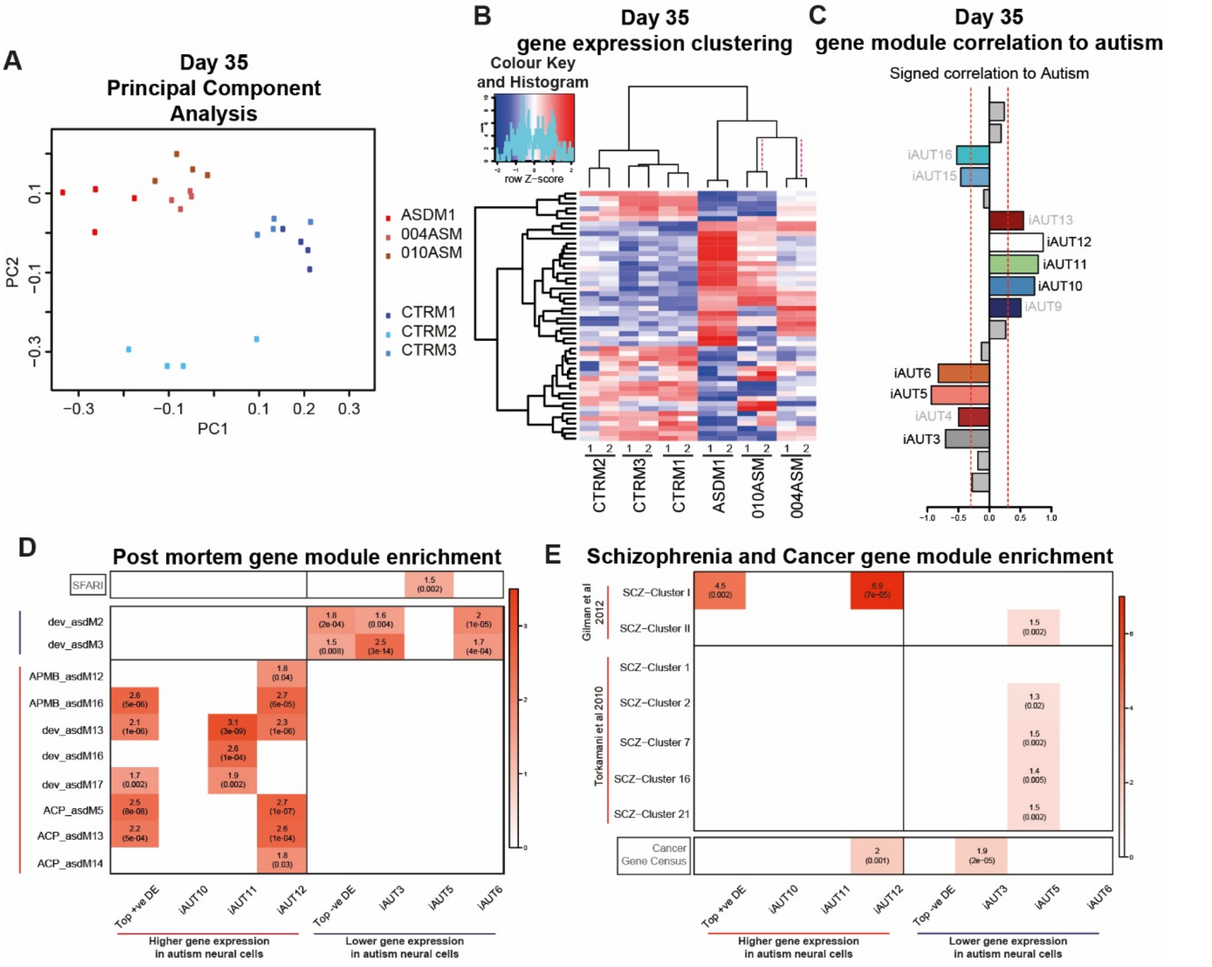

Supplementary Figure S3: RNA-sequencing analysis of day 35 cortical neurons. (A) Principal component analysis showing distinct transcriptomic profiles of autism and typical day35 cells. (B) Gene count from iPSC biological replicates (1, 2) of individual participants and clustering based on Z-scores of top 50 differentially expressed genes. Clustering of 010ASM, 004ASM indicated using magenta dashed lines. (C) WGCNA reveals 11 gene modules significantly correlated to autism (top 3 positively correlated and top 3 negatively correlated modules enrichment shown; greyed module enrichment not shown). (D) Gene module enrichment reveals positively correlated (red) modules are enriched in corresponding positively correlated *post-mortem* gene modules, while negatively correlated (blue) modules are enriched in negatively correlated *post-mortem* gene modules. (E) Gene modules do not show sufficient enrichment in *post-mortem* gene modules from schizophrenia studies or cancer gene sets.

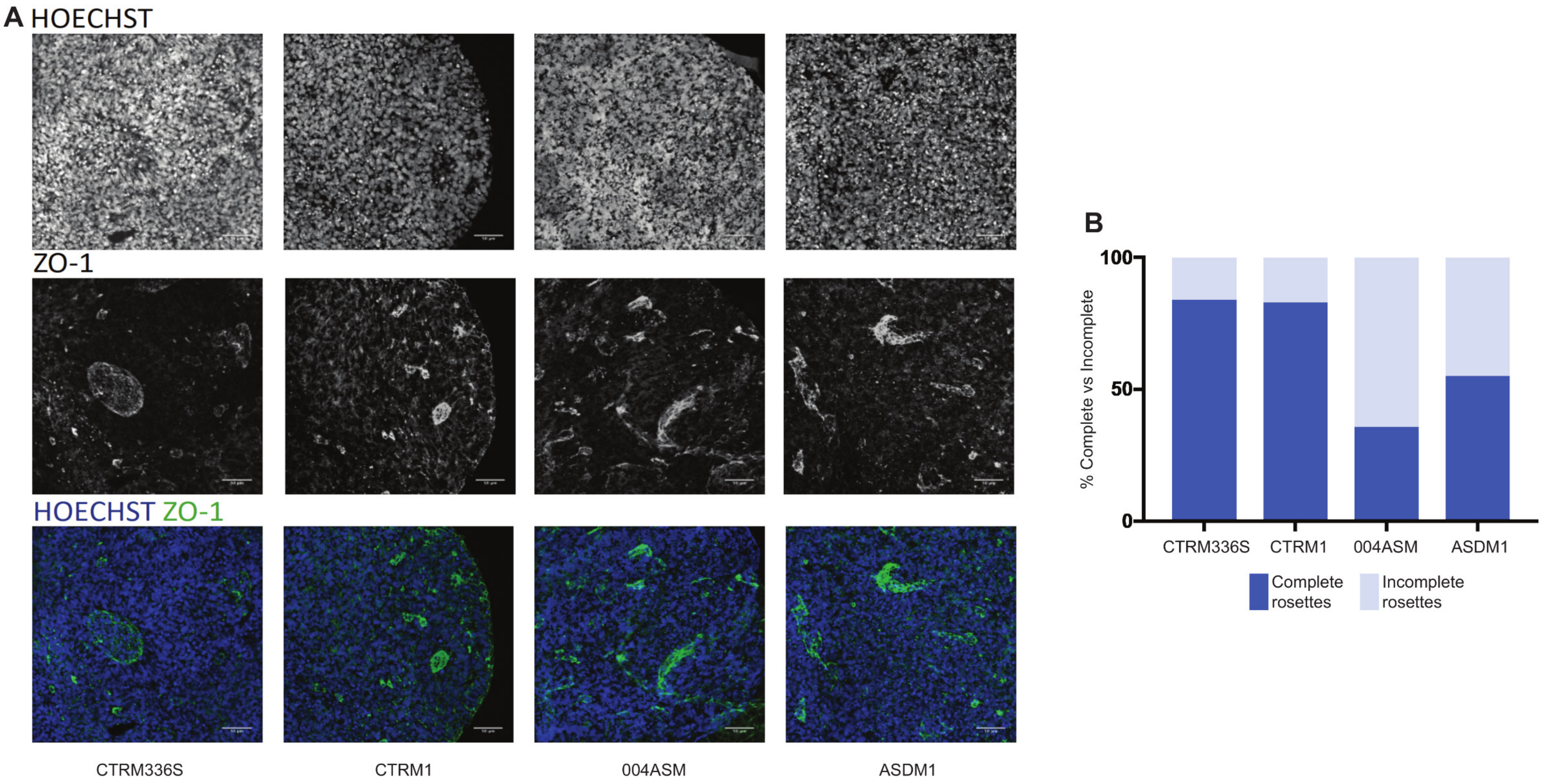

Supplementary Figure S4: Day 7 3D spheroids from contro-l and autism-iPSCs demonstrate deficiency in neural rosette formation. (A) Day 30 spheroids were immunostained for ZO1 (green) and Hoescht (Blue). **(B)** Percent complete and incomplete rosettes.

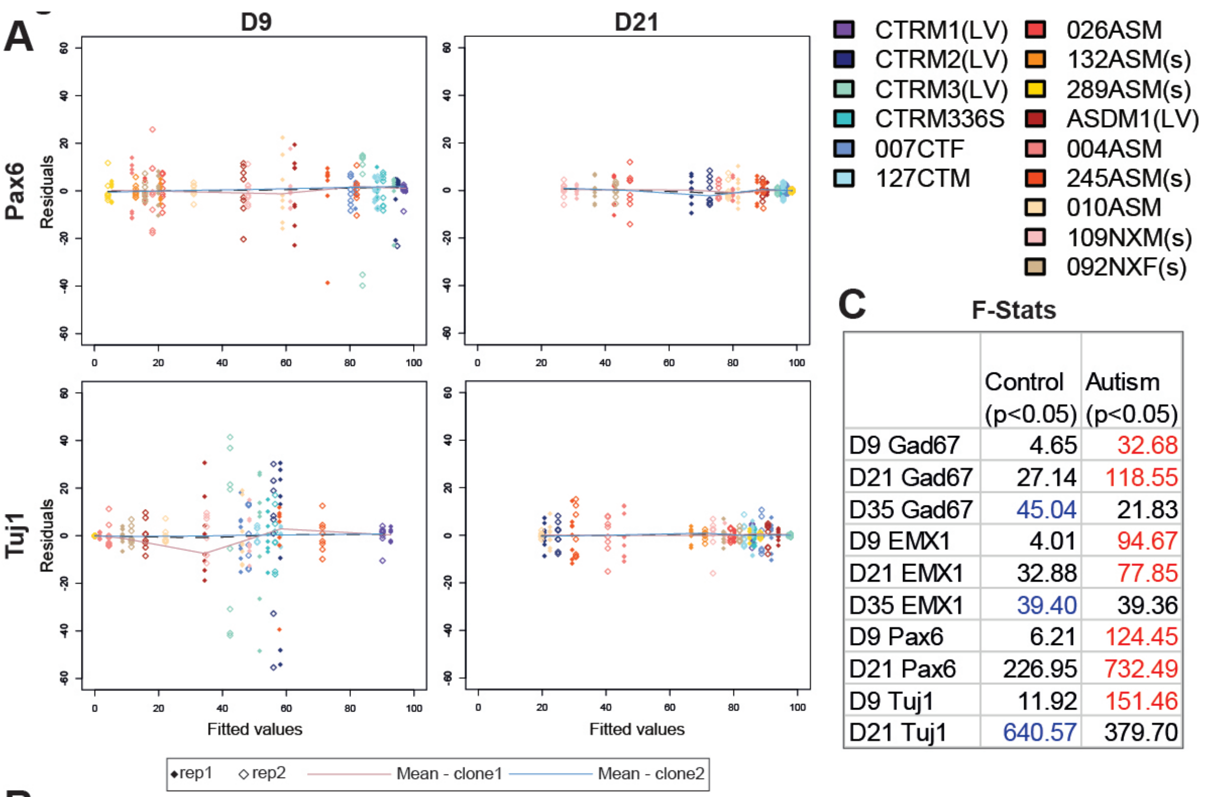

C

F-Stats

	Control (p<0.05)	Autism (p<0.05)
D9 Gad67	4.65	32.68
D21 Gad67	27.14	118.55
D35 Gad67	45.04	21.83
D9 EMX1	4.01	94.67
D21 EMX1	32.88	77.85
D35 EMX1	39.40	39.36
D9 Pax6	6.21	124.45
D21 Pax6	226.95	732.49
D9 Tuj1	11.92	151.46
D21 Tuj1	640.57	379.70

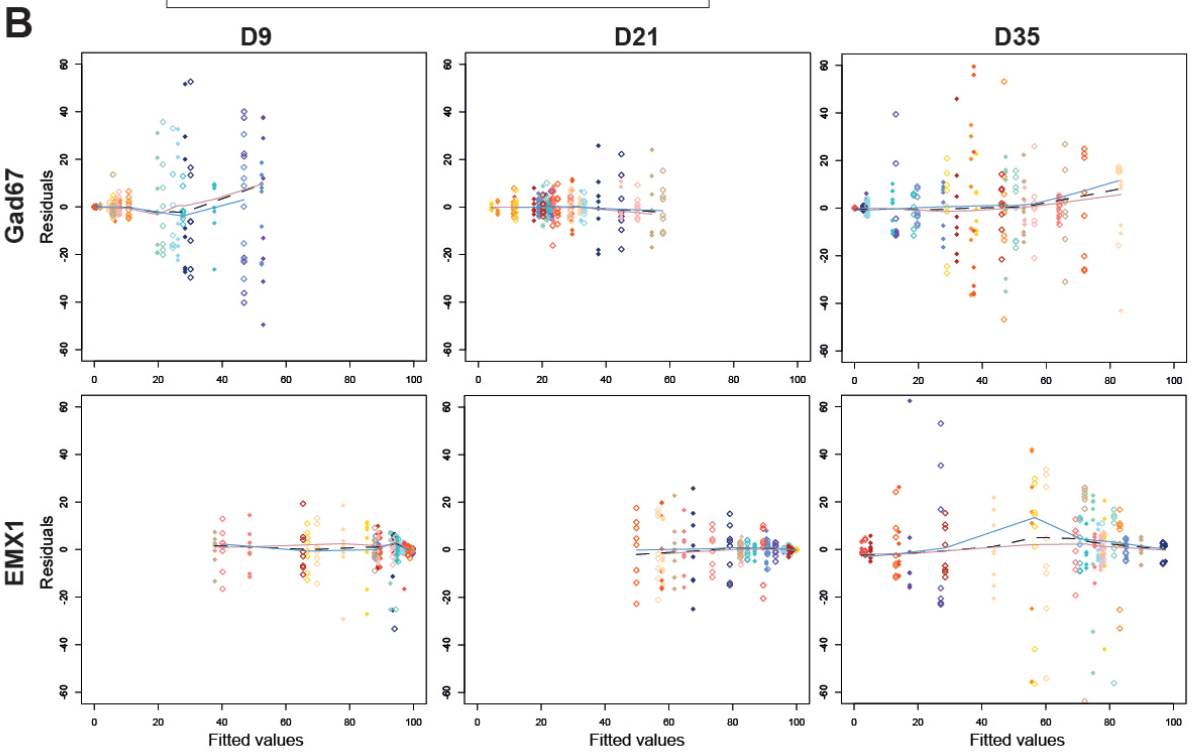

Supplementary Figure S5: ANOVA plots to demonstrate changes in variance of data points due to clones from individual participants. (A) Two-way ANOVA (Lines~Cells+Clones) Residuals vs Fitted values plots when observing cortical differentiation. **(B)** Two-way ANOVA (Lines~Cells+Clones) Residuals vs Fitted values plots demonstrate spread of values of individual data points across all samples when observing dorsal vs ventral forebrain differentiation. **(C)** F-values show degree of variance within the control and autism groups. All parameters measured show significant variance ($p < 0.05$) across both groups, and 7 out of 10 parameters show greater variance in the autism group.

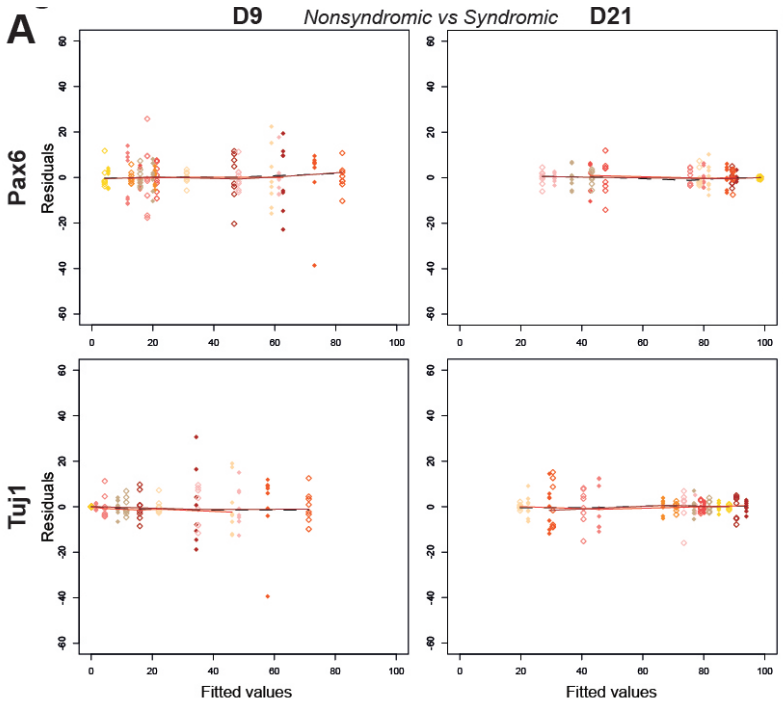

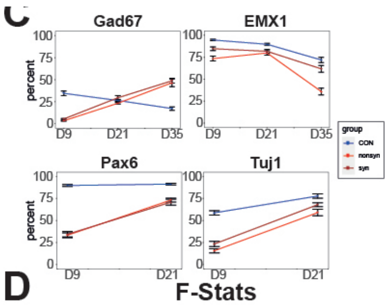

D F-Stats

	Nonsyndromic (p<0.05)	Syndromic (p<0.05)
D9 Gad67	84.22	23.63891
D21 Gad67	29.15	160.81
D35 Gad67	71.81	3.86
D9 EMX1	104.86	87.67
D21 EMX1	77.98	99.91
D35 EMX1	88.62	22.31
D9 Pax6	53.78	297.39
D21 Pax6	244.87	2222.23
D9 Tuj1	70.44	260.08
D21 Tuj1	604.37	279.91

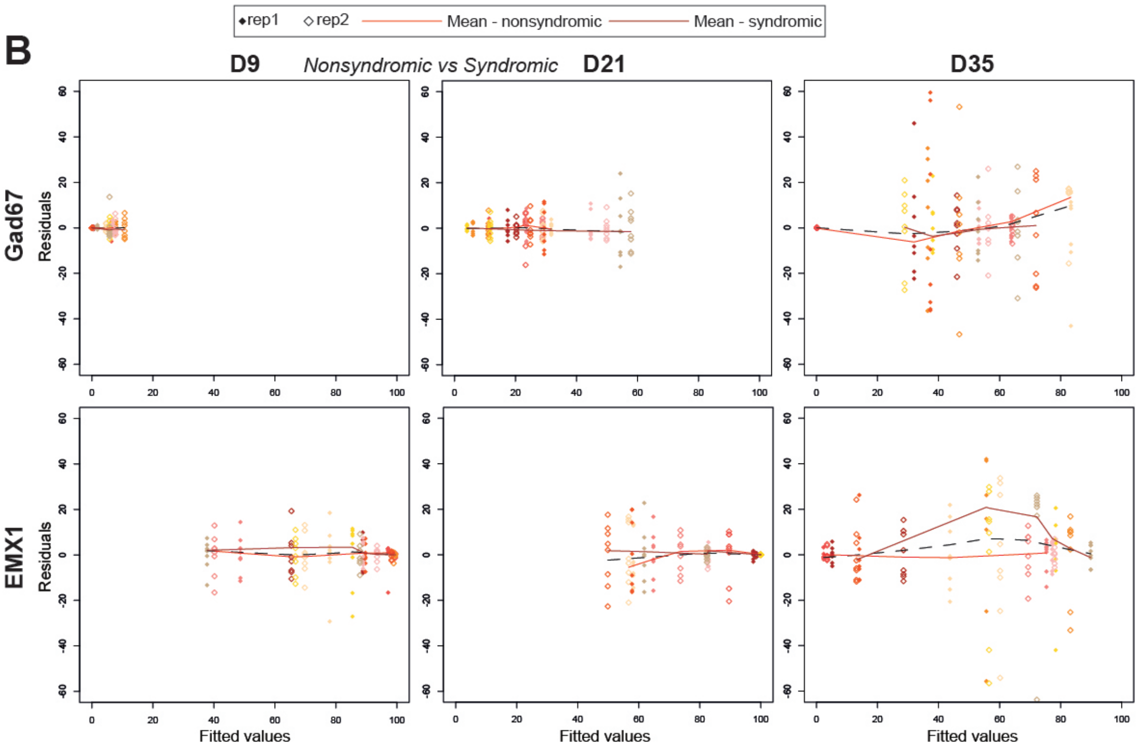

Supplementary Figure S6: ANOVA plots to demonstrate changes in variance of data points due to type of diagnosis (non-syndromic vs syndromic) from individual participants. (A) Two-way ANOVA (Lines~Cells+Syndromic) Residuals vs Fitted values plots when observing non-syndromic vs syndromic cortical differentiation in autistic samples. **(B)** Two-way ANOVA (Lines~Cells+Syndromic) Residuals vs Fitted values plots demonstrate spread of values of individual data points across all autistic samples when observing non-syndromic vs syndromic dorsal vs ventral forebrain differentiation. **(C)** Mean values of % positive cells in controls, nonsyndromic and syndromic autism plotted over time. **(D)** F-values

show degree of variance within the non-syndromic and syndromic autism groups. All parameters measured show significant variance ($p < 0.05$) across both groups, and each group shows greater variance compared to the other in equal number of parameters, 5 out of 10.

**Table S1: General information on participants**									
Donor No.	Cohort	Ethics	iPSC ID	Autism Diagnosis	Comorbidity	Head size	Sex	Age (years)	Ethnicity
1	EU-AIMS LEAP	PINDS	ASDM1	Yes		Normal	M	34	Caucasian
2	EU-AIMS LEAP	PINDS	004ASM	Yes		Normal	M	26	Caucasian
3	EU-AIMS LEAP	PINDS	010ASM	Yes	ADHD/Depression	Normal	M	27	Caucasian
4	EU-AIMS LEAP	PINDS	026ASM	Yes		Normal	M	16	Caucasian
5	GOS-ICH	PINDS	289ASM	Yes		Normal	M	19	Caucasian
6	GOS-ICH	PINDS	132ASM	Yes	ADHD	Normal	M	8	Caucasian
7	BBGRE	PINDS	245ASM	Yes		Normal	M	15	Caucasian
8	BBGRE	PINDS	092NXF	Yes		Normal	F	10	Caucasian
9	BBGRE	PINDS	109NXM	Yes		Microcephaly	M	9	Caucasian
1	StemBANCC	PINDS	CTRM1	No		Normal	M	58	Caucasian
2	StemBANCC	PINDS	CTRM2	No		Normal	M	33	Caucasian
3	StemBANCC	PINDS	CTRM3	No		Normal	M	37	Caucasian
4	StemBANCC	PINDS	CTRM336S	No		Normal	M	37	Caucasian
5	EU-AIMS LEAP	PINDS	127CTM	No		Normal	M	56	Caucasian
6	EU-AIMS LEAP	PINDS	007CTF	No		Normal	F	27	Caucasian

Table S2: Diagnosis details of autistic participants

Donor no.	Cohort	iPSC ID	Autism Diagnosis	ADOS				ADI-R				
				Social Interaction	Communication	Imagination	Stereotypic behaviour	PD_B	PD_C	PD_CS	PD_D	BDI
1	EU-AIMS LEAP	ASDM1	Yes	4	4	1	3					22
2	EU-AIMS LEAP	004ASM	Yes	8	4	1	2	25	19	NA	4	3
3	EU-AIMS LEAP	010ASM	Yes	5	5	1	2	3	3	NA	3	25
4	EU-AIMS LEAP	026ASM	Yes	9	10	NA	9	23	22	NA	1	
5	GOS-ICH	289ASM	Yes; syndromic	3	3	0	1	9	5.7	2.3	1.3	
6	GOS-ICH	132ASM	Yes; syndromic	10	9	NA	9	15.8	14	7.5	5	
7	BBGRE*	245ASM	Yes; syndromic									
8	BBGRE*	092NXF	Yes; syndromic									
9	BBGRE*	109NXM	Yes; syndromic									

*Autism diagnosis was confirmed by referring physicians for BBGRE cohort participants. No further information available.

**Table S3: Genetic data of autistic participants**			
Donor no.	Cohort	iPSC ID	Known Deletions/Duplications/SNVs
1	EU-AIMS LEAP	ASDM1	
2	EU-AIMS LEAP	004ASM	
3	EU-AIMS LEAP	010ASM	
4	EU-AIMS LEAP	026ASM	
5	GOS-ICH	289ASM	SNV- Chr19:41759516; C->T; AXL gene; STOP_GAINED; LoF, Synaptic transmission (Schizophrenia). CNV VOUS: 8q21.12 to q21.13 del - 8;79,886,962- 80,149,513.
6	GOS-ICH	132ASM	CNV Clinical abnormality - 1p21.3 del - 1:96,953,361-97,711,563 (758,201bp, DPYD, PTBP2). VOUS: 3q25.33 to q26.1 del- 3:160,629,302- 160,727,203 (97,900bp, PPM1L); 8p11.23 Dup- 8:37,124,969-37,186,580 (61,611bp); 15q26.1 dup- 15:92,818,328-92,875,717 (57,388bp); 17q24.1 del- 17:63,288,669-63,375,080 (86,410bp);
7	BBGRE	245ASM	Complete duplication of CNTN6; paternal x3 chr3:4,354,703-4,532,449 (partail dup SETMAR; complete duplication of SUMF1)
8	BBGRE	092NXF	Paternally inherited duplication in long arms of chromosome 1 – likely to be benign – 1q21.1 (144,679,874 – 145,747,269) x3. De novo deletion of ~200kb in short arm of chromosome 2 – 2p16.3 (50,806,991 – 51,013,685) x1
9	BBGRE	109NXM	Maternally inherited deletion ~60kb in short arm of chromosome 2 – 2p16.3 (50,888,852 – 50,947,729) x1

**Table S4: Reprogramming method and iPSC validation**					
**Donor no.**	**iPSC ID**	**Reprogramming method**	**hiPSC validation**	**Diagnosis**	**Diagnosis type**
1	ASDM101	Constitutive Polycistronic Lentivirus Reprogramming Kit*	ICC; cytoSNP	Autism	Nonsyndromic
	ASDM108	Constitutive Polycistronic Lentivirus Reprogramming Kit*	ICC; cytoSNP	Autism	Nonsyndromic
2	004ASM01	CytoTune-iPS Sendai Reprogramming Kit	ICC; cytoSNP	Autism	Nonsyndromic
	004ASM09	CytoTune-iPS Sendai Reprogramming Kit	ICC; cytoSNP	Autism	Nonsyndromic
3	010ASM05	CytoTune-iPS Sendai Reprogramming Kit	ICC; cytoSNP	Autism	Nonsyndromic
	010ASM06	CytoTune-iPS Sendai Reprogramming Kit	ICC; cytoSNP	Autism	Nonsyndromic
4	026ASM03	CytoTune-iPS Sendai Reprogramming Kit	ICC; cytoSNP	Autism	Nonsyndromic
	026ASM02	CytoTune-iPS Sendai Reprogramming Kit	ICC; cytoSNP	Autism	Nonsyndromic
5	132ASM02	CytoTune-iPS Sendai Reprogramming Kit	ICC; cytoSNP	Autism	Nonsyndromic
	132ASM01	CytoTune-iPS Sendai Reprogramming Kit	ICC; cytoSNP	Autism	Nonsyndromic
6	289ASM03	CytoTune-iPS Sendai Reprogramming Kit	ICC; cytoSNP	Autism	Nonsyndromic
	289ASM01	CytoTune-iPS Sendai Reprogramming Kit	ICC; cytoSNP	Autism	Nonsyndromic
7	245ASM02	CytoTune-iPS Sendai Reprogramming Kit	ICC; cytoSNP	Autism	Syndromic
	245ASM04	CytoTune-iPS Sendai Reprogramming Kit	ICC; cytoSNP	Autism	Syndromic

8	109NXM04	CytoTune-iPS Sendai Reprogramming Kit	ICC; cytoSNP	Autism	Syndromic
	109NXM03	CytoTune-iPS Sendai Reprogramming Kit	ICC; cytoSNP	Autism	Syndromic
9	092NXF04	CytoTune-iPS Sendai Reprogramming Kit	ICC; cytoSNP	Autism	Syndromic
	092NXF09	CytoTune-iPS Sendai Reprogramming Kit	ICC; cytoSNP	Autism	Syndromic
**Controls iPSCs**					
1	CTRM104	Constitutive Polycistronic Lentivirus Reprogramming Kit*	ICC; cytoSNP		
	CTRM111	Constitutive Polycistronic Lentivirus Reprogramming Kit*	ICC; cytoSNP		
2	CTRM205	Constitutive Polycistronic Lentivirus Reprogramming Kit*	ICC; cytoSNP		
	CTRM242	Constitutive Polycistronic Lentivirus Reprogramming Kit*	ICC; cytoSNP		
3	CTRM315	Constitutive Polycistronic Lentivirus Reprogramming Kit*	ICC; cytoSNP		
	CTRM322	Constitutive Polycistronic Lentivirus Reprogramming Kit*	ICC; cytoSNP		
4	CTRM336S	CytoTune-iPS Sendai Reprogramming Kit	ICC; cytoSNP		

	CTRM337S	CytoTune-iPS Sendai Reprogramming Kit	ICC; cytoSNP		
5	127CTM04	CytoTune-iPS Sendai Reprogramming Kit	ICC; cytoSNP		
	127CTM10	CytoTune-iPS Sendai Reprogramming Kit	ICC; cytoSNP		
6	007CTF10	CytoTune-iPS Sendai Reprogramming Kit	ICC; cytoSNP		
	007CTF01	CytoTune-iPS Sendai Reprogramming Kit	ICC; cytoSNP		

*Note: The constitutive polycistronic lentivirus kit uses a single vector which has a negligible risk of insertional mutagenesis and viral reactivation

**Table S5: Fluorescent thresholds for antibodies used in cell type analysis**				
**Antibody**	**Supplier**	**Cat. No.**	**Dilution**	**Fluorescent intensity threshold (a.u.)**
Pax6	BioLegend	901301	1:300	3500
Tuj1	BioLegend	801201	1:500	2500
Emx1	ThermoFisher	PA5-35373	1:100	4000
Gad67	Abcam	ab26116	1:1000	600
TBR1	Abcam	ab31940	1:200	1000
CD44	R&D Systems	MAB7045	1:50	600
LMX1A	Abcam	ab139726	1:100	1000
FOXA2	Invitrogen	701698	1:300	3000

Table S6. Differentially expressed genes and module assignments

See Excel File

**Table S7: Morphological details of Neural Rosettes**						
**Participant ID**	**Average diameter (mm)**	**T-test ($p=1\times10^{-22}$)**	**(mm)**	**Mean rosettes number (%)**	**T-test ($p=6\times10^{-11}$)**	**(%)**
CTRM1	0.070855781	**Mean (control)**	0.0742113	1.714263246	**Mean (control)**	1.87176
CTRM2	0.070123668			0.980711145		
CTRM3	0.090845642			1.785184347		
CTRM336S	0.066494935			0.65878026		
007CTF	0.07566226			2.981324875		
127CTM	0.071285499			3.110279078		
026ASM	0	**Mean (autism)**	0.04042801	0	**Mean (autism)**	6.52623
132ASM	0.053815315			11.14960454		
289ASM	0.049887732			4.940428375		
ASDM1	0.055942962			5.255250823		
004ASM	0			0		
245ASM	0.057726738			3.7669185		
010ASM	0.070457893			2.453844441		
109NXM	0.055274512			12.55877341		
092NXF	0.060892681			5.558788098		

Supplementary Table S8: Variability in data points due to clones. Data points showing significant variation due to clones highlighted in red.

	Clones F-stat					
	Combined	*p-val*	Control	*p-val*	Autism	*p-val*
D9 Gad67	0.279	*0.598*	0.478	*0.4912*	1.568	*0.2129*
D21 Gad67	13.355	*0.0003*	1.554	*0.216*	14.505	*0.0002*
D35 Gad67	5.435	*0.0207*	4.817	*0.03*	0.634	*0.428*
D9 EMX1	0.269	*0.605*	0.025	*0.8751*	0.291	*0.591*
D21 EMX1	1.635	*0.202*	1.239	*0.2688*	0.663	*0.417*
D35 EMX1	1.095	*0.2965*	0.515	*0.475*	0.603	*0.4388*
D9 Pax6	11.116	*0.001*	0.598	*0.441*	13.65	*0.0003*
D21 Pax6	0.683	*0.41*	1.473	*0.2283*	0.062	*0.8035*
D9 Tuj1	4.19	*0.0419*	0.496	*0.483*	10.69	*0.0014*
D21 Tuj1	0.099	*0.7538*	0.04	*0.842*	0.06	*0.8072*
D35 TBR1	0.091	*0.763*	1.459	*0.231*	1.244	*0.267*

Supplementary References

1. Aasen T, Izpisua Belmonte JC (2010): Isolation and cultivation of human keratinocytes from skin or plucked hair for the generation of induced pluripotent stem cells. *Nat Protoc*. 5:371-382.
2. Takahashi K, Tanabe K, Ohnuki M, Narita M, Ichisaka T, Tomoda K, et al. (2007): Induction of pluripotent stem cells from adult human fibroblasts by defined factors. *Cell*. 131:861-872.
3. Shi Y, Kirwan P, Livesey FJ (2012): Directed differentiation of human pluripotent stem cells to cerebral cortex neurons and neural networks. *Nat Protoc*. 7:1836-1846.
4. Fedele S, Collo G, Behr K, Bischofberger J, Muller S, Kunath T, et al. (2017): Expansion of human midbrain floor plate progenitors from induced pluripotent stem cells increases dopaminergic neuron differentiation potential. *Sci Rep*. 7:6036.
5. Kriks S, Shim JW, Piao J, Ganat YM, Wakeman DR, Xie Z, et al. (2011): Dopamine neurons derived from human ES cells efficiently engraft in animal models of Parkinson's disease. *Nature*. 480:547-551.
6. Pasca AM, Sloan SA, Clarke LE, Tian Y, Makinson CD, Huber N, et al. (2015): Functional cortical neurons and astrocytes from human pluripotent stem cells in 3D culture. *Nat Methods*. 12:671-678.
7. Carpenter AE, Jones TR, Lamprecht MR, Clarke C, Kang IH, Friman O, et al. (2006): CellProfiler: image analysis software for identifying and quantifying cell phenotypes. *Genome Biol*. 7:R100.
8. Dobin A, Davis CA, Schlesinger F, Drenkow J, Zaleski C, Jha S, et al. (2013): STAR: ultrafast universal RNA-seq aligner. *Bioinformatics*. 29:15-21.
9. Li H, Handsaker B, Wysoker A, Fennell T, Ruan J, Homer N, et al. (2009): The Sequence Alignment/Map format and SAMtools. *Bioinformatics*. 25:2078-2079.
10. Hartley SW, Mullikin JC (2015): QoRTs: a comprehensive toolset for quality control and data processing of RNA-Seq experiments. *BMC Bioinformatics*. 16:224.
11. Anders S, Pyl PT, Huber W (2015): HTSeq--a Python framework to work with high-throughput sequencing data. *Bioinformatics*. 31:166-169.
12. Hansen KD, Irizarry RA, Wu Z (2012): Removing technical variability in RNA-seq data using conditional quantile normalization. *Biostatistics*. 13:204-216.
13. Langfelder P, Horvath S (2008): WGCNA: an R package for weighted correlation network analysis. *BMC Bioinformatics*. 9:559.
14. Parikshak NN, Luo R, Zhang A, Won H, Lowe JK, Chandran V, et al. (2013): Integrative functional genomic analyses implicate specific molecular pathways and circuits in autism. *Cell*. 155:1008-1021.
15. Parikshak NN, Swarup V, Belgard TG, Irimia M, Ramaswami G, Gandal MJ, et al. (2016): Genome-wide changes in lncRNA, splicing, and regional gene expression patterns in autism. *Nature*.
16. Csardi G, Nepusz T (2006): The igraph software package for complex network research. *InterJournal, Complex Systems*. 1695:1-9.

17. Pasca SP, Portmann T, Voineagu I, Yazawa M, Shcheglovitov A, Pasca AM, et al. (2011): Using iPSC-derived neurons to uncover cellular phenotypes associated with Timothy syndrome. *Nat Med*. 17:1657-1662.

18. Zhang Y, Sloan SA, Clarke LE, Caneda C, Plaza CA, Blumenthal PD, et al. (2016): Purification and Characterization of Progenitor and Mature Human Astrocytes Reveals Transcriptional and Functional Differences with Mouse. *Neuron*. 89:37-53.

19. Zambon AC, Gaj S, Ho I, Hanspers K, Vranizan K, Evelo CT, et al. (2012): GO-Elite: a flexible solution for pathway and ontology over-representation. *Bioinformatics*. 28:2209-2210.

20. Loth E, Charman T, Mason L, Tillmann J, Jones EJH, Wooldridge C, et al. (2017): The EU-AIMS Longitudinal European Autism Project (LEAP): design and methodologies to identify and validate stratification biomarkers for autism spectrum disorders. *Mol Autism*. 8:24.

21. Ahn JW, Dixit A, Johnston C, Ogilvie CM, Collier DA, Curran S, et al. (2013): BBGRE: brain and body genetic resource exchange. *Database (Oxford)*. 2013:bat067.

22. Malmgren H, Sahlen S, Wide K, Lundvall M, Blennow E (2007): Distal 3p deletion syndrome: detailed molecular cytogenetic and clinical characterization of three small distal deletions and review. *Am J Med Genet A*. 143A:2143-2149.
